# Supplementary material for: EEG-based AI-BCI wheelchair advancement: Transformer-based learning with motor imagery for brain computer interface
Source: Biol Methods Protoc. 2026 Jul 8;11(1):bpag039. doi: 10.1093/biomethods/bpag039 (PMC13401820; doi:10.1093/biomethods/bpag039)
Supplement: bpag039_Supplementary_Data [file bpag039_supplementary_data.docx]

**Section 1: Hardware Configuration and Deployment Metrics**

Table S1 presents the hardware configuration of the Raspberry Pi system used in the experimentation.

**Table S1: Hardware Configuration**

| **Component** | **Available / Total** |
| --- | --- |
| RAM | 1845.6 MB |
| Swap | 1845.0 MB |
| Disk | 14.03 GB |

Table S2 presents the results of 20 inference runs, reporting inference time, CPU usage, and RAM utilization during model deployment.

**Table S2: Inference performance metrics on Raspberry Pi (20 runs)**

| **Row No.** | **Inference Time (s)** | **CPU Usage (%)** | **RAM Used (MB)** |
| --- | --- | --- | --- |
| 1 | 0.0190 | 15.0 | 250.8 |
| 2 | 0.0176 | 15.0 | 257.6 |
| 3 | 0.0138 | 19.5 | 257.7 |
| 4 | 0.0138 | 17.1 | 257.8 |
| 5 | 0.0137 | 15.0 | 257.8 |
| 6 | 0.0140 | 15.4 | 257.8 |
| 7 | 0.0141 | 15.0 | 257.8 |
| 8 | 0.0138 | 17.5 | 257.8 |
| 9 | 0.0141 | 17.5 | 257.8 |
| 10 | 0.0143 | 19.0 | 257.8 |
| 11 | 0.0141 | 17.5 | 257.8 |
| 12 | 0.0140 | 15.4 | 257.8 |
| 13 | 0.0143 | 15.0 | 257.8 |
| 14 | 0.0140 | 15.4 | 257.8 |
| 15 | 0.0140 | 17.1 | 257.8 |
| 16 | 0.0144 | 17.5 | 257.8 |
| 17 | 0.0142 | 17.1 | 257.8 |
| 18 | 0.0144 | 17.1 | 257.8 |
| 19 | 0.0142 | 15.4 | 257.8 |
| 20 | 0.0140 | 15.0 | 257.8 |
| **Average** | **0.0145** | **16.43** | **257.4** |

**Section 2: Receiver Operating Characteristic and Precision–Recall Curves**

This section presents the Receiver Operating Characteristic (ROC) and Precision–Recall (PRC) area under the curve for all evaluated models. The plots show one-versus-rest class-wise curves and the macro-averaged curve, providing a comprehensive evaluation of model performance across varying decision thresholds, particularly under class imbalance conditions.


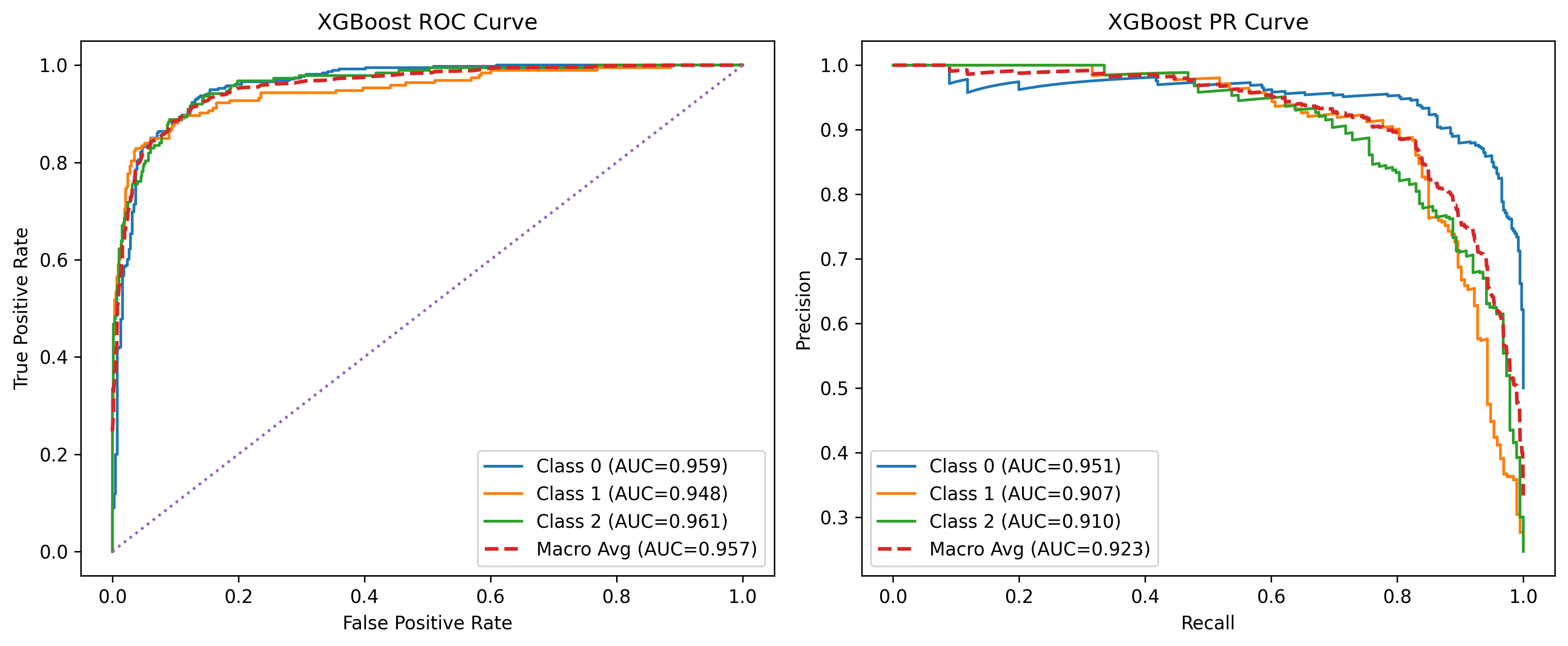


**Fig S1**. Performance curves for the XGBoost model: (a) ROC and (b) PRC


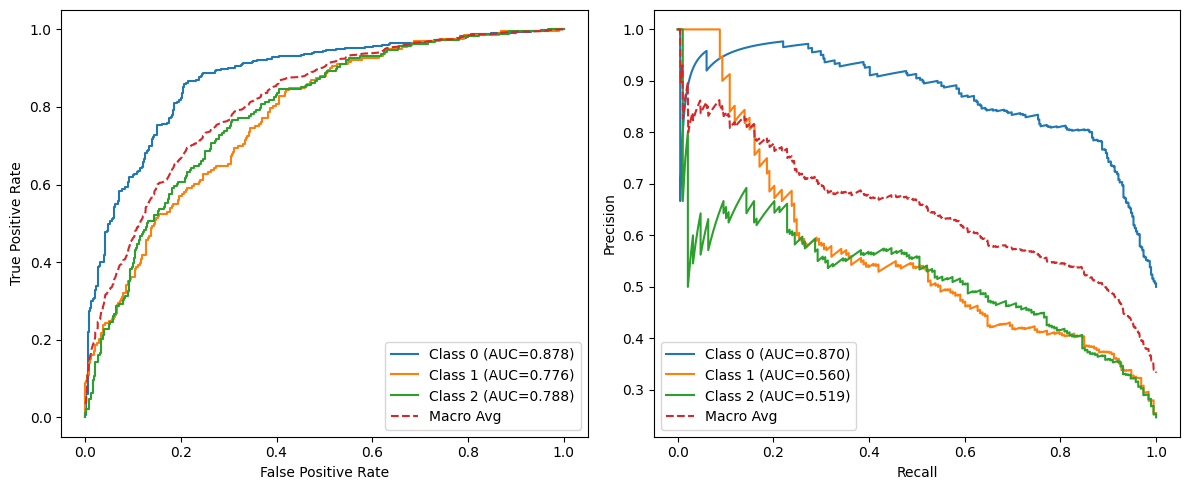


**Fig S2**. Performance curves for the EEGNet model: (a) ROC and (b) PRC


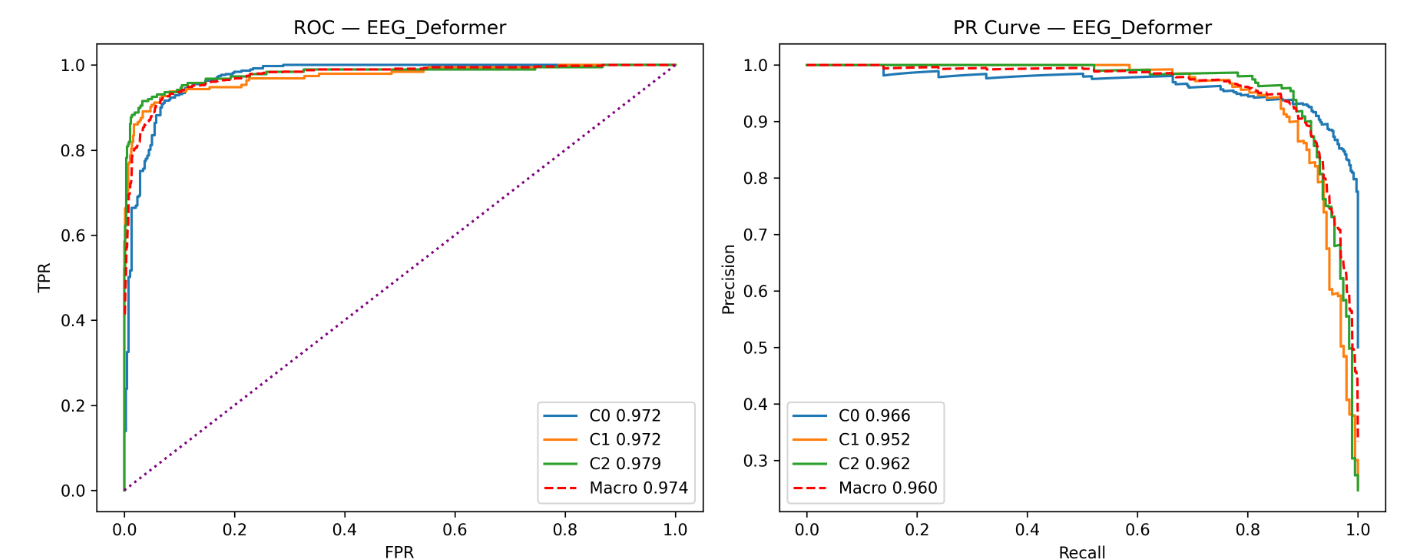


**Fig S3**. Performance curves for the EEG-Deformer model: (a) ROC and (b) PRC


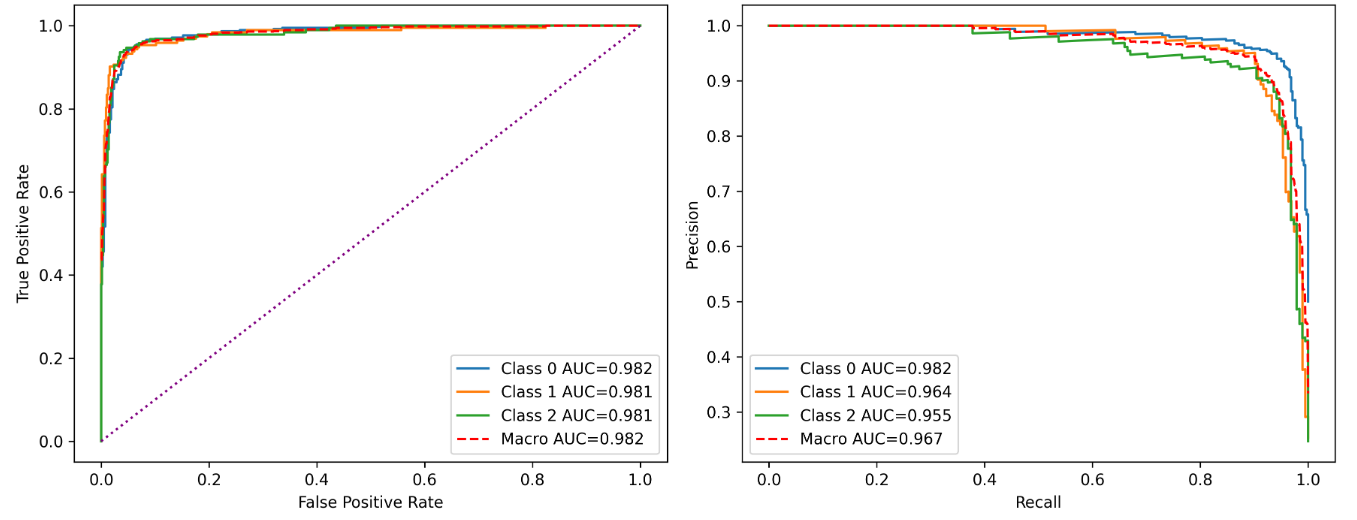


**Fig S4:** Performance curves for the TFormerEEG (a) ROC and (b) PRC
